# The Spitting Habit

**Published:** 1896-10

**Authors:** W. D. Howells


ARTICLE III.

### THE SPITTING HABIT.

There have been recent expressions of feeling about a certain gross sin against decency which I think must have had the concurrence of all right-thinking or at-all-thinking people. This sin is so common among us that the shame of it has attached to the American name in the mind of every alien who has visited us, and of many who have merely heard of us. I am talking, of course, of our loathsome vice of spitting in public places.

The early observers of our manners supposed this was an effect of the tobacco-chewing habit, and they hoped it would disappear with that. But tobacco-chewing, in the North at least, is almost as obsolete as snuff-taking; and yet the other offensive seems as rife as ever. It is so bad that if one thinks of it, one must keep one's eyes well lifted from the pavement, or suffer a distress in walking abroad which would not afflict one in any other civilized country. In our own country I have an impression that the habit is worse in New York than elsewhere, but perhaps it is the character of our paving that renders it peculiarly obvious, though this would not account for its disgusting conspicuity at every turn. What makes it so maddenly offensive is that it is the habit of people who would not dream of offering you an offence if they once thought of it. A quite well-dressed savage will commit this sort of nuisance a dozen times a day, and pass on ignorant of the qualms that he has inflicted, and in the full conviction that he is incapable of a filthy outrage. Yet he is really a savage in what he has done, and the fact that he has done it thoughtlessly accounts for him rather than excuses him. The well-bred man, the gentleman that every American wishes to be held, is pledged to the thought of others in everything, their rights, their feelings, and if he forgets them he is so far false to his ideal of conduct. But a nasty habit of any sort is something even worse than this in the man who indulges it; it is a shabby and shameful act of oppressing which the witnesses and victims cannot resist. If a man spits in your presence, you cannot right yourself or restore the tone of your nerves by telling him he is a dirty fellow; it would not avail if you did, and perhaps in other things he is not a dirty fellow; at any rate, it is not the custom to be frank with such offenders, and you must suffer in silence. His habit unhappily *is* the custom, especially the American custom, as all sidewalks, common stairways and public passages bear sickening proof, and he may turn your stomach without breaking the peace; but if you reproved him for it in adequate terms, you must be guilty of something actionable. He has injured and insulted you, and you have no redress. You have no recourse but to civilize him, and this, I understand from some late utterances in the press, is what the ladies are going to attempt.

Women are the chief sufferers by any breach of good manners or good morals, and they are peculiarly the sufferers from this habit, which is a breach of both. They can hardly go into the street without bringing home some evidence of it on their garments, or enter a public conveyance without incurring the risk of a nauseous pollution from it. The savage who has put upon them this cruel indignity, this vile and cowardly injury, may be far away when it happens to them, and probably is so. But if by chance he should be at hand or within reach of their angry eyes, it is proposed that they shall fix him with an incinerating stare, and then lift their gaze significantly to the placards whtch appeal in many public places to gentlemen against spitting on the floor.

The measure does not seem very drastic, but it will do for a beginning, Perhaps its efficiency might be promoted by multiplying the placards mentioned, and relanguaging them. They ought to be put up not only in all conveyances and halls, stairways and vestibules, but at every street corner and on every lamp post. There ought to be let into the sidewalks, at intervals of half a block, tablets of brass or marble, and instead of entreating gentlemen not to spit in a given place, these ought to be mandatory and objurgatory in the highest degree. To call such offenders gentlemen, and request their polite forbearance in a matter of such mere elementary decency, is too comical. I suppose I shall hardly live to see some offender of this sort arrested, and given his choice of cleaning up his filth or going to jall, but posterity may have this pleasure.—W. D. HOWELLS in *Harper's Weekly*.

